# Suppression of Contraction Raises Calcium Ion Levels in the Heart of Zebrafish Larvae

**DOI:** 10.3390/bios14050219

**Published:** 2024-04-27

**Authors:** Antonio Martinez-Sielva, Manuel Vicente, Jussep Salgado-Almario, Aarón Garcia-Blazquez, Beatriz Domingo, Juan Llopis

**Affiliations:** Physiology and Cell Dynamics Group, Instituto de Biomedicina de la Universidad de Castilla-La Mancha, Facultad de Medicina de Albacete, Universidad de Castilla-La Mancha, C/Almansa 14, 02006 Albacete, Spain; antonio.martinez34@alu.uclm.es (A.M.-S.); manuel.vicente@uclm.es (M.V.); jussep.salgado@uclm.es (J.S.-A.); aaron.garcia@uclm.es (A.G.-B.)

**Keywords:** ratiometric Ca^2+^ biosensor, aequorin, zebrafish heart, cardiac contraction, blebbistatin, tnnt2a morpholino, excitation–contraction coupling, mechano-electric coupling

## Abstract

Zebrafish larvae have emerged as a valuable model for studying heart physiology and pathophysiology, as well as for drug discovery, in part thanks to its transparency, which simplifies microscopy. However, in fluorescence-based optical mapping, the beating of the heart results in motion artifacts. Two approaches have been employed to eliminate heart motion during calcium or voltage mapping in zebrafish larvae: the knockdown of cardiac troponin T2A and the use of myosin inhibitors. However, these methods disrupt the mechano-electric and mechano-mechanic coupling mechanisms. We have used ratiometric genetically encoded biosensors to image calcium in the beating heart of intact zebrafish larvae because ratiometric quantification corrects for motion artifacts. In this study, we found that halting heart motion by genetic means (injection of *tnnt2a* morpholino) or chemical tools (incubation with *para*-aminoblebbistatin) leads to bradycardia, and increases calcium levels and the size of the calcium transients, likely by abolishing a feedback mechanism that connects contraction with calcium regulation. These outcomes were not influenced by the calcium-binding domain of the gene-encoded biosensors employed, as biosensors with a modified troponin C (Twitch-4), calmodulin (mCyRFP1-GCaMP6f), or the photoprotein aequorin (GFP-aequorin) all yielded similar results. Cardiac contraction appears to be an important regulator of systolic and diastolic Ca^2+^ levels, and of the heart rate.

## 1. Introduction

Optical mapping of membrane potential and Ca^2+^ in the heart of zebrafish larvae with synthetic indicators or gene-encoded biosensors has proved to be a valuable technique for studying physiological and disease mechanisms [1]. However, quantification of the fluorescence in defined regions of interest (ROIs) over the atrium or ventricle is prone to motion artifacts, as the beating heart moves with respect to the fixed ROIs. Therefore, either heart motion is suppressed, or computer algorithms are used to correct these artifacts. Different approaches have been used to stop the heart. One involves the knockdown of cardiac troponin T2A by the microinjection of a *tnnt2a* antisense morpholino oligonucleotide (MO) into embryos [2]. This procedure disrupts cardiac contraction and blood flow as soon as the heart tube is formed, phenocopying the silent heart (*sih*) mutations. Another approach is to use inhibitors of actin–myosin interaction like blebbistatin or its analogs [3,4]. Although these methods have allowed the investigation of electrical excitation and Ca^2+^ dynamics in zebrafish [5,6], the mechano-electric and mechano-mechanic coupling mechanisms [7], which adjust the heart rate and stroke volume to the changing physiological needs, are abrogated.

Mechano-electrical coupling refers to the process by which mechanical changes in cardiomyocytes lead to changes in their electrical activity. This mechanism is crucial for maintaining proper cardiac rhythm and function. When the heart contracts, it generates mechanical forces that can affect its electrical behavior, and conversely, changes in electrical activity influence contraction. Ca^2+^ ions play a central role in excitation–contraction coupling, the relationship between electrical signaling on the plasma membrane (an action potential) and cardiac contraction. The use of *tnnt2a* MO or blebbistatin to uncouple contraction has been shown not to affect the periodical electrical excitation nor the occurrence of cardiac Ca^2+^ transients (CaTs) [2,8]. However, because of the close link between Ca^2+^ dynamics and contraction, we hypothesized that stopping the heart in Ca^2+^-mapping experiments might affect Ca^2+^ homeostasis in a more subtle way that may have remained unnoticed.

By using ratiometric gene-encoded Ca^2+^ biosensors, we have recently imaged Ca^2+^ in the beating hearts of intact zebrafish larvae [9,10]. Since motion affects the two emission bandwidths of these biosensors, ratioing the respective fluorescence images cancels out most motion artifacts [11], whereas the emission ratio remains sensitive to Ca^2+^. In this work, Ca^2+^ levels and contraction were assessed simultaneously in the heart of zebrafish control larvae, of larvae injected with a *tnnt2a* MO, or incubated with *para*-aminoblebbistatin (PAB). Three different gene-encoded Ca^2+^ biosensors were used to measure Ca^2+^ dynamics, yielding similar results. We show that suppressing contraction, regardless of the means used, results in bradycardia, alters Ca^2+^ homeostasis and raises Ca^2+^ levels.

## 2. Methods

### 2.1. Fish Husbandry and Morpholino Microinjection

The zebrafish lines used in this work were *Tg(myl7:Twitch-4)* (ZFIN ID: ZDB-TGCONSTRCT-231106-2), *Tg(myl7:mCyRFP1-GCaMP6f)*, *Tg(myl7:GFP-Aequorin)* (ZFIN ID: ZDB-TGCONSTRCT-231023-1) and wild-type AB fish [9,10,12]. Fish were maintained in the Center for Animal Experimentation of the Albacete School of Medicine with a 14/10 h light/dark cycle. Synchronously fertilized zebrafish eggs were obtained following standard procedures and kept in E3 medium (5 mM NaCl, 0.17 mM KCl, 0.33 mM MgSO_4_, 0.33 mM CaCl_2_, pH 7.4 in double-distilled H_2_O) at 28.5 °C. The antisense MO targeted against troponin T2A (*tnnt2a*) (5′-CATGTTTGCTCTGATCTGACACGCA-3′) (ZFIN ID: ZDB-MRPHLNO-060317-4) [2] (Gene Tools LLC, Philomath, OR, USA) was injected (2 ng) into one-cell-stage fertilized eggs to knockdown *tnnt2a* expression in larvae. A standard control MO designed to block a human beta-globin intron (5′-CCTCTTACCTCAGTTACAATTTATA-3′) was also injected as a negative control [13]. Finally, another group of fertilized eggs was injected with double-distilled H_2_O.

### 2.2. Mounting of Larvae for Fluorescence Microscopy

Non-anesthetized 3- and 5-day-post-fertilization (dpf) larvae were embedded in 100 μL of 0.3% low-melting-point agarose in E3 medium preheated to 42 °C on a 96-well plate (for fluorescence) or an 8-well plate (for bioluminescence) with square wells and a flat bottom (ibidi, Gräfelfing, Germany). Larvae were gelled in a ventral side position during the agarose solidification, followed by the addition of 100 μL of E3 medium. Then, larvae were held under a microscope at 28 °C for 30 min to obtain a stable heart rate. Where indicated, larvae were treated with 75 µM of *para*-aminoblebbistatin (PAB) (Motorpharma, Budapest, Hungary), a myosin inhibitor, for 2 h before mounting for microscopy. For the PAB washout experiments, untreated larvae (control) and larvae incubated in PAB for 1 or 2 h were imaged; two additional groups of larvae incubated for 2 h with PAB had the drug washed out for 2 or 4 h before imaging.

### 2.3. Fluorescence Imaging

Fluorescence and transmitted light images were acquired from the heart of 3 and 5 dpf *Tg(myl7:Twitch-4)*, and 3 dpf *Tg(myl7:mCyRFP1-GCaMP6f)* larvae. We used a wide-field fluorescence microscope (DMIRE-2, Leica Microsystems, Wetzlar, Germany) equipped with an sCMOS camera (ORCA-Flash 4.0, Hamamatsu Photonics, Hamamatsu, Japan), controlled by Aquacosmos 2.6 software (Hamamatsu Photonics, Hamamatsu, Japan). Larvae were imaged in a chamber incubator (PeCon GmbH, Erbach, Germany) at 28 °C for 5 s at an acquisition rate of 50 frames/s with continuous excitation from an LED source (Lambda TLED+, Sutter Instrument, Novato, CA, USA). A 440AF21 nm bandpass filter was used to excite Twitch-4 and a 470/40 ET nm filter was used for mCyRFP1-GCaMP6f excitation (both filters from Chroma, Bellows Falls, VT, USA). A 10× air objective (HC PlanApo 0.45 NA, Leica Microsystems, Wetzlar, Germany) was used for all imaging experiments. The emission from the two fluorescent proteins in each biosensor was captured simultaneously with an image splitter (W-View Gemini, Hamamatsu Photonics, Hamamatsu, Japan), dividing the camera field into two halves, giving two emission channels. For Twitch-4, the beamsplitter 509-FDi01 was used to separate the fluorescent emissions (483/32 nm and 542/27 nm bandpass filters). For mCyRFP1-GCaMP6f, the beamsplitter H560 LPXR was used with 525/50 nm and 620/60 nm bandpass filters (all filters were from Semrock, Rochester, NY, USA). Images were acquired at a 16-bit depth with 2 × 2 binning; the spatial resolution was 1.45 μm × 1.45 μm/pixel.

### 2.4. Bioluminescence Imaging

We acquired bioluminescence images from the hearts of 3 dpf *Tg(myl7:GFP-Aequorin)* larvae. A stock of diacetyl *h*-coelenterazine at 7.4 mM was prepared in dimethyl sulfoxide, and 5 µL aliquots were stored at −80 °C and used at a 50 µM final concentration. Aequorin reconstitution was carried out as previously described [12]. PAB at 75 µM was added to the bath during the 2 h coelenterazine incubation period of the aequorin reconstitution protocol. Bioluminescence images were obtained with a custom-built low-light microscope (components were from Thorlabs GmbH, Bergkirchen, Germany) equipped with an EM-CCD camera (512 × 512 pixels, EMC9100-13, Hamamatsu Photonics, Hamamatsu, Japan). A 4× CFI Plan Apochromat Lambda air objective (Nikon, Tokio, Japan) was used as the tube lens, and an air 20× Nikon CFI Plan Apochromat Lambda (0.75 NA) as the objective. The combination of these lenses results in a magnification of 5 × (f tube lens/f objective lense). Illumination for transmitted light was achieved with an LED lamp and the whole microscope was housed in a light-tight box to maintain darkness. Larvae were kept at 28 °C during imaging. Bioluminescence images were acquired continuously at a 16-bit depth with 4 × 4 binning, 255 EM gain, and a rate of 1 frame/s to obtain a time-averaged luminescence signal. With this configuration, the spatial resolution of the images was 12.8 µm × 12.8 µm/pixel.

### 2.5. Image Processing and Data Analysis

Processing and analysis of fluorescence images were performed with the custom software Ratioscope [14], written in IGOR Pro software (WaveMetrics, Lake Oswego, OR, USA), which is available at https://zenodo.org/records/11059482 (accessed on 24 April 2024) under DOI 10.5281/zenodo.11059481. Pixel shift in the emission channels was corrected and the ratio *FRET image/donor image* (for Twitch-4) or *Ca^2+^-sensitive image/reference image* (for mCyRFP1-GCaMP6f) was calculated on a pixel-by-pixel basis for each time point. Regions of interest (ROIs) were manually drawn over the atrium and the ventricle external profiles in diastole. The ratio value for an ROI was calculated as the average of all the pixels’ values weighted by the average intensity of the two channels. As the ratio value of pixels whose fluorescence is close to the background can reach infinite values, pixels whose values were smaller than the *minimum displayed ratio*/4 or larger than the *maximum displayed ratio* × 4 were clipped. To reduce the noise in some ratio traces, a Savitzky–Golay smoothing filter was applied. From the obtained ratio traces, several kinetic parameters were automatically calculated: systolic Ca^2+^ (the highest ratio in the cardiac cycle), diastolic Ca^2+^ (the lowest ratio in the cardiac cycle), CaT amplitude (ΔRatio, systolic − diastolic ratio), and the frequency of atrial CaT (min^−1^), as previously described [9]. Data shown for each larva were calculated as the average of all the cardiac cycles within 5 s of continuous recording (250 images). The ventricular area was measured by drawing an ROI over the ventricle wall in systole (end-systolic area) and diastole (end-diastolic area) as previously described [10]. The fractional area change (FAC) was calculated as follows:

FAC = (end-diastolic area − end-systolic area)/end-diastolic area.

GFP-aequorin bioluminescence images were analyzed as reported [12]. ROIs were drawn over the ventricle, avoiding the atrial region, and the luminescence signal (in relative light units, RLU) was transformed into *luminescence rate* (*L*, in counts s^−1^). The *total counts* (*Ltotal*, in RLU) were obtained as the summatory of all the luminescence values throughout the experiment. *Lconsumed* was calculated as the sum of all *L* values from time zero to any given time point. *Lconsumed* represents the amount of aequorin that was already spent at each time point. Lastly, *Lmax* was calculated as *Ltotal* − *Lconsumed* at each time point. *Lmax* represents the available aequorin at each time point and is the sum of detected counts from that time point to the end of the experiment. Finally, the value *L*/*Lmax* is proportional to the Ca^2+^ levels at each time point.

Transmitted light images were processed and analyzed in ImageJ [15], and the yolk area was measured by drawing an ROI over the yolk.

### 2.6. Statistics

Statistical analysis was performed with GraphPad Prism 9 (Graphpad Software, Boston, MA, USA) and Igor Pro (WaveMetrics, Lake Oswego, OR, USA). The number of larvae (*n*) and the statistical tests applied are indicated in each figure caption. The Shapiro–Wilk test was used to test for the normality of each dataset. Differences between two groups were tested using the unpaired Student’s *t*-test for parametric data or the Mann–Whitney test for non-parametric data. Comparisons between more than two groups were analyzed by one-way ANOVA with Tukey’s multiple comparisons post-test for parametric data, or Kruskal–Wallis with Dunn’s multiple comparisons post-test for non-parametric data. Comparisons of nominal variables between groups were analyzed using the χ^2^ test. Correlations between variables were assessed by linear regression and the calculation of the coefficient of determination (R^2^). Data are shown as the mean ± SD. A *p* < 0.05 was considered statistically significant, and significances are indicated in the figures as * for *p* < 0.05, ** *p* < 0.01, *** *p* < 0.001, and **** *p* < 0.0001 for parametric statistics, and ^x^ for *p* < 0.05, ^xx^ *p* < 0.01, ^xxx^ *p* < 0.001, and ^xxxx^ *p* < 0.0001 for non-parametric statistics.

## 3. Results

### 3.1. Morphological and Functional Alterations in tnnt2a Morphant Larvae

**Figure 1:**
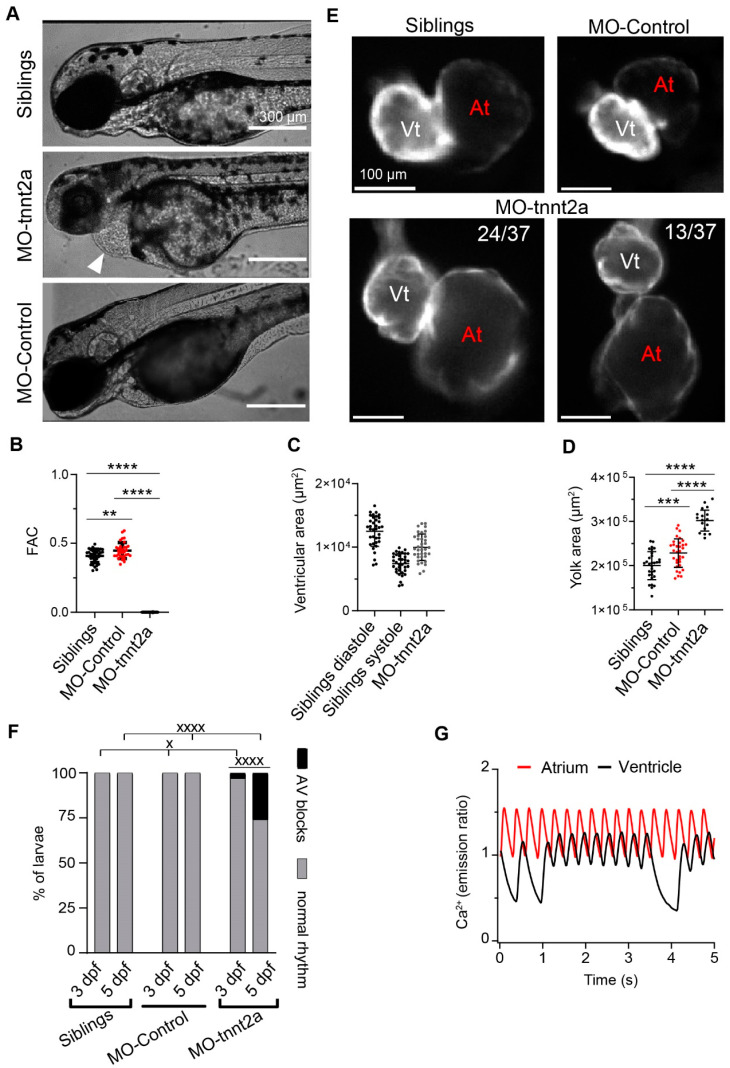
Morphological and functional alterations in 3 dpf *tnnt2a* morphant larvae in *Tg(myl7:Twitch-4)* zebrafish. (**A**) Pericardial edema (arrow) was observed in *tnnt2a* morphants compared to their siblings. (**B**) Fractional area change (FAC, see Section 2) measured in the ventricle shows that the *tnnt2a* morpholino completely abolishes heart contraction. (**C**) *Tnnt2a* morphant larvae had a ventricular area in between that of sibling larvae in systole and diastole (*n* = 36 sibling, *n* = 37 MO *tnnt2a*). (**D**) Yolk area of siblings (*n* = 31), larvae injected with control MO (*n* = 33), and those injected with MO *tnnt2a* (*n* = 17). (**E**) Morphology of atrium and ventricle of representative sibling, control morphant, and *tnnt2a* morphant larvae. The number of larvae presenting a particular phenotype in *tnnt2a* MO larvae is shown. (**F**) Percentage of sibling, control morphant, and *tnnt2a* morphant larvae displaying AV blocks at 3 and 5 dpf. (**G**) Atrial and ventricular Ca^2+^ traces of a representative 3 dpf *Tg(myl7:mCyRFP1-GCaMP6f)* larva showing atrioventricular conduction blocks (i.e., a drop in ventricular CaT). Statistical analysis in C was performed using a one-way ANOVA test with Tukey’s multiple comparisons post-test. Statistical analysis in E was performed using a χ^2^ test. Data are shown as the mean ± SD ** for *p* < 0.01, *** for *p* < 0.001 and **** *p* < 0.0001 for parametric statistics; ^x^ for *p* < 0.05 and ^xxxx^ for *p* < 0.0001 for non-parametric statistics). The scale bars in (**A**,**D**) indicate 300 µm and 100 µm, respectively.

*Tnnt2a* MO was injected into fertilized eggs at the one-cell stage. In addition, an MO targeting a human beta-globin intron (control MO) was used as a control of unspecific effects, and H_2_O was employed as a control of the injection procedure. The silent heart (*sih*) phenotype [2] was successfully generated in *tnnt2a* morphants. Unlike in sibling larvae, there was pericardial edema (Figure 1A), and the heart of 3 dpf *tnnt2a* morphants was arrested (Figure 1B and Videos S1 and S2). In *tnnt2a* morphants, there was a wide dispersion of ventricular areas compared with their siblings in systole and diastole (Figure 1C). As the yolk is gradually consumed throughout larval development, we used the decrease in the yolk area as a marker of developmental progression. An enlarged yolk area in *tnnt2a* morphants, and, to a lesser degree, in control morphant larvae, was observed (Figure 1D), suggesting a developmental lag. We also found an abnormal shape of the atrium and atrioventricular canal (Figure 1E), probably related to the pericardial edema, in one-third of *tnnt2a* MO-injected larvae. We also monitored atrial and ventricular excitation, detected as the CaT frequency in each chamber, since heart beating was absent. *Tnnt2a* morphant larvae exhibited atrioventricular blocks, which were not found in sibling or control morphant larvae (3 dpf χ^2^ = 6.6061, *p* < 0.05; 5 dpf χ^2^ = 55.11, *p* < 0.0001, control MO vs. *tnnt2a* MO) (Figure 1F,G). Such blocks became more frequent at 5 dpf (χ^2^ = 20.37, *p* < 0.0001, 3 vs. 5 dpf). This is a further indication of the severe alterations in heart development and function induced by the *tnnt2a* MO [2].

### 3.2. Downregulation of tnnt2a Induced Aberrant Ca^2+^ Dynamics in 3 dpf Larvae

**Figure 2:**
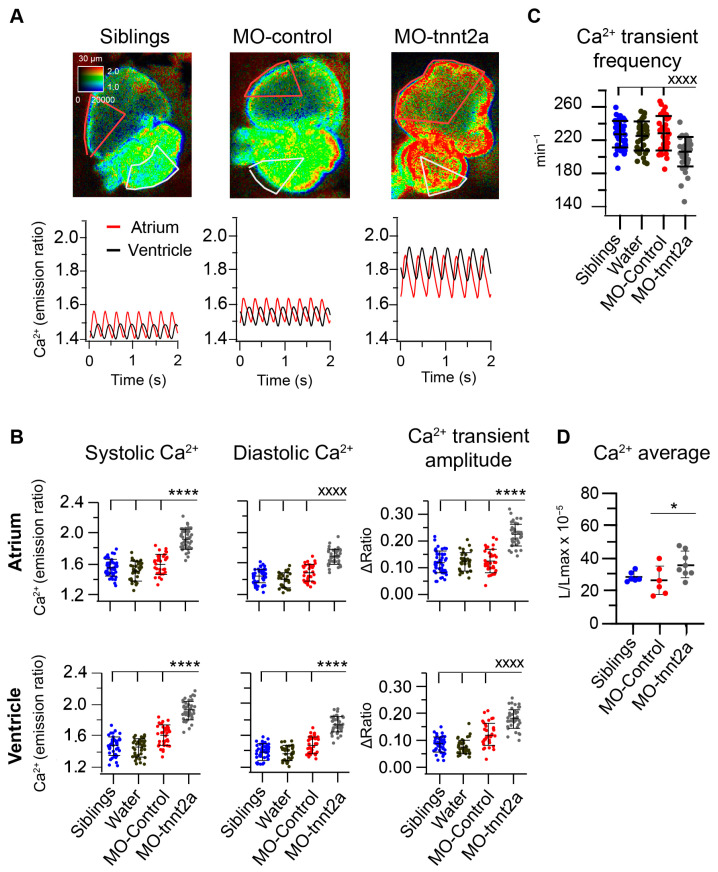
Increased Ca^2+^ levels, Ca^2+^ transient amplitude, and bradycardia in 3 dpf larvae injected with *tnnt2a* MO. (**A**) Ratiometric images of hearts in pseudocolor and their corresponding atrial (red) and ventricular (black) Ca^2+^ traces from representative 3 dpf *Tg(myl7:Twitch-4)* sibling and morphant larvae. Images from siblings and MO controls show the ventricular mechanical systole. The calibration square shows the distance in µm (horizontal length), whereas the hue codes for the emission ratio, and intensity codes for the fluorescence intensity. (**B**) Atrial and ventricular systolic Ca^2+^ (Twitch-4 emission ratio), diastolic Ca^2+^, and Ca^2+^ transient amplitude in sibling (*n* = 29), water-injected (*n* = 39), control morphant (*n* = 29), and *tnnt2a* morphant (*n* = 41) larvae. (**C**) Atrial CaT frequency (min^−1^) in these larvae. (**D**) Time-averaged Ca^2+^ levels (L/L_max_) measured by bioluminescence of 3 dpf *Tg(myl7:GFP-aequorin)* sibling (*n* = 6), control morphant (*n* = 6), and *tnnt2a* morphant (*n* = 8) larvae. Diacetyl *h*-coelenterazine was used as the aequorin substrate. Statistical analysis was performed using a one-way ANOVA test with Tukey’s multiple comparisons post-test or Kruskal–Wallis with Dunn’s multiple comparisons post-test, for parametric or non-parametric statistics, respectively. In (**B**,**C**), the mean of MO-tnnt2a morphants was compared with the mean of the other groups. Data are shown as the mean ± SD (* for *p* < 0.05 and **** *p* < 0.0001 for parametric statistics; ^xxxx^ for *p* < 0.0001 for non-parametric statistics).

The *tnnt2a* MO has been shown to uncouple contraction from excitation [2,6]. To determine whether *tnnt2a* downregulation affects Ca^2+^ dynamics, we performed in vivo Ca^2+^ imaging in 3 dpf *Tg(myl7:Twitch-4)* larvae expressing the Ca^2+^ biosensor Twitch-4 in the heart [9]. *Tnnt2a* morphant larvae showed higher atrial and ventricular Ca^2+^ levels in systole and diastole, as well as increased CaT amplitude, compared to sibling, water-injected, and control morphant larvae (Figure 2A,B). Control morphant hearts showed a modest increase in Ca^2+^ levels, and water-injected larvae had no change compared to siblings. Additionally, *tnnt2a* morphants exhibited a lower frequency of atrial CaT in comparison to their siblings, larvae injected with water, or control morphant larvae (Figure 2C).

**Figure 3:**
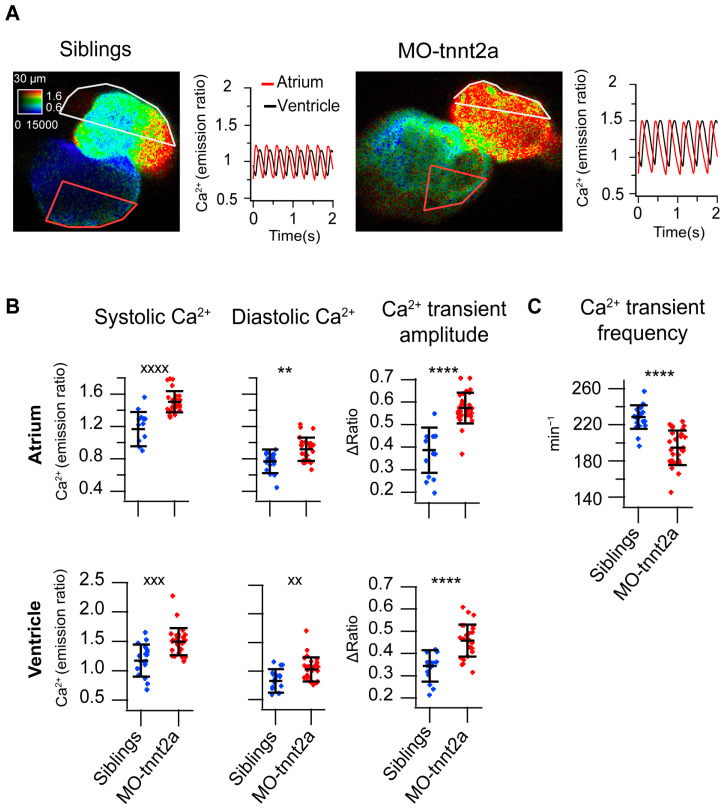
Increased Ca^2+^ levels, Ca^2+^ transient amplitude, and bradycardia in 3 dpf *Tg(myl7:mCyRFP1-GCaMP6f)* larvae injected with *tnnt2a* MO. (**A**) Ratiometric images of hearts and atrial and ventricular Ca^2+^ traces from representative 3 dpf *Tg(myl7: mCyRFP1-GCaMP6f)* sibling and *tnnt2a* morphant larvae. The image of siblings shows the mechanical systole of the ventricle. (**B**) Atrial and ventricular systolic Ca^2+^ (mCyRFP1-GCaMP6f emission ratio), diastolic Ca^2+^, and Ca^2+^ transient amplitude in sibling (*n* = 23) and *tnnt2a* morphant (*n* = 28) larvae. (**C**) Atrial CaT frequency (min^−1^) in these larvae. Statistical analysis was performed using an unpaired Student’s *t*-test and the Mann–Whitney test for non-parametric data. Data are shown as the mean ± SD (** for *p* < 0.01 and **** *p* < 0.0001 for parametric statistics; ^xx^ for *p* < 0.01, ^xxx^ *p* < 0.001, and ^xxxx^ *p* < 0.0001 for non-parametric statistics).

To rule out any non-specific effect of Twitch-4, a biosensor with a mutated troponin-C as the Ca^2+^-binding domain, two other methods were used to image heart Ca^2+^ levels. We used cardioluminescence of GFP-aequorin expressed in the heart in the *Tg(myl7:GFP-Aequorin)* zebrafish line [12]. *Tnnt2a* morphants displayed higher time-averaged ventricular Ca^2+^ levels than control morphant larvae (Figure 2D). Finally, we also used the transgenic line *Tg(myl7:mCyRFP1-GCaMP6f)* [10]. The systolic and diastolic Ca^2+^ levels and the CaT amplitude increased in the *tnnt2a* morphant larvae (Figure 3A,B), whereas the atrial CaT frequency decreased (Figure 3C), confirming the findings obtained with Twitch-4.

Taken together, these results show that the downregulation of *tnnt2a* disrupted excitation–contraction coupling, induced bradycardia, and caused a rise in Ca^2+^ levels and CaT amplitude in the zebrafish heart. These effects were measured with biosensors bearing three different Ca^2+^-sensitive proteins: a modified troponin C (in Twitch-4), calmodulin (in mCyRFP1-GCaMP6f), and the photoprotein aequorin (in GFP-aequorin), which rules out non-specific effects caused by the expression of a particular Ca^2+^-binding domain.

### 3.3. Pharmacological Myosin Inhibition with Para-Aminoblebbistatin Alters Ca^2+^ Dynamics in 3 dpf Larvae

**Figure 4:**
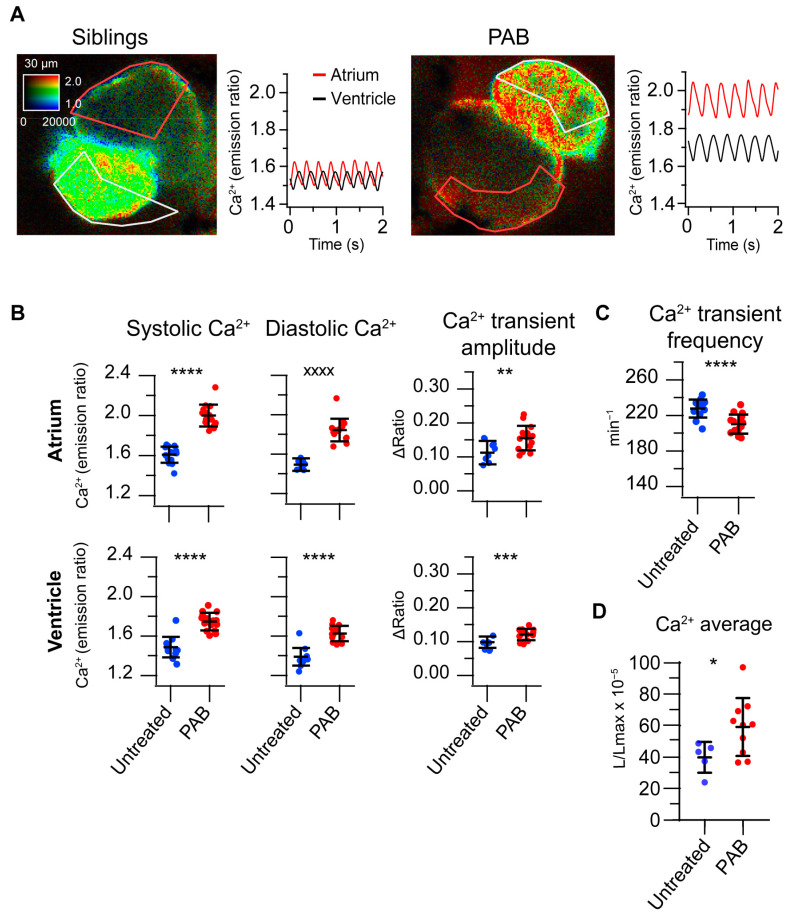
Suppression of heart contraction with *para*-aminoblebbistatin raises Ca^2+^ levels, Ca^2+^ transient amplitude, and induces bradycardia in 3 dpf larvae. (**A**) Ratiometric images of hearts and atrial and ventricular Ca^2+^ traces from representative 3 dpf *Tg(myl7:Twitch-4)* untreated larvae and larvae preincubated with *para*-aminoblebbistatin (PAB, 75 µM) for 2 h. Image of the untreated larva shows the mechanical systole of the ventricle. (**B**) Atrial and ventricular systolic Ca^2+^ (Twitch-4 emission ratio), diastolic Ca^2+^, and Ca^2+^ transient amplitude in untreated larvae (*n* = 15), and in larvae preincubated with PAB (*n* = 15). (**C**) Atrial CaT frequency (min^−1^) in these larvae. (**D**) Time-averaged Ca^2+^ levels (L/L_max_) measured by bioluminescence of 3 dpf *Tg(myl7:GFP-aequorin)* untreated larvae (*n* = 5), and in larvae preincubated with 75 µM PAB for 2 h (*n* = 10). Diacetyl *h*-coelenterazine was used as the aequorin substrate. Statistical analysis was performed using an unpaired Student’s *t*-test for parametric data and the Mann–Whitney test for non-parametric data. Data are shown as the mean ± SD (* for *p* < 0.05, ** *p* < 0.01, *** *p* < 0.001, and **** *p* < 0.0001 for parametric statistics, and ^xxxx^ *p* < 0.0001 for non-parametric statistics).

To determine whether the alterations in Ca^2+^ dynamics observed in *tnnt2a* morphants were attributable to the developmental abnormalities of the heart caused by the *tnnt2a* MO or rather because of stopping the heart, we used an acute pharmacological approach. The myosin inhibitor blebbistatin and its derivates like PAB have been shown to disrupt cardiac contraction in zebrafish larvae [6,9,16,17] and in isolated hearts from mammalian models [18,19]. The incubation of 3 dpf *Tg(myl7:Twitch-4)* zebrafish larvae with 75 µM PAB for 2 h suppressed heart contraction almost completely (Video S3). Systolic and diastolic Ca^2+^ levels and CaT amplitude increased in both the atrium and ventricle of PAB-treated larvae (Figure 4A,B), and the atrial CaT frequency decreased (Figure 4C), replicating the effects observed in the *tnnt2a* morphants. In addition, cardioluminescence experiments in 3 dpf *Tg(myl7:GFP-Aequorin)* zebrafish confirmed these findings: larvae treated with PAB had higher time-averaged Ca^2+^ levels in the ventricle than untreated larvae (Figure 4D). These results suggest that the raised Ca^2+^ levels and bradycardia observed in *tnnt2a* morphants and in PAB-incubated larvae were due to the lack of heart motion.

**Figure 5:**
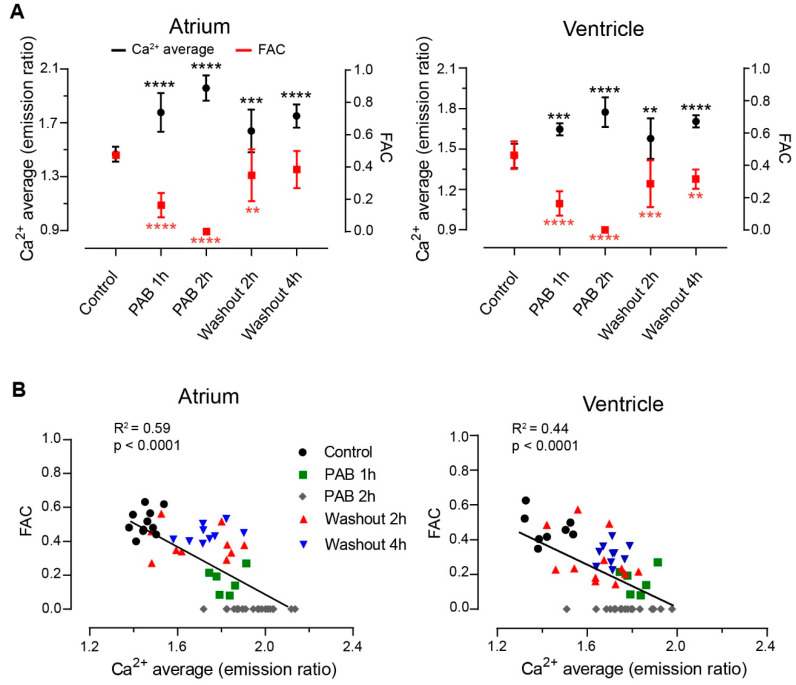
Incubation and washout of *para*-aminoblebbistatin in 3 dpf *Tg(myl7:Twitch-4)* zebrafish larvae. (**A**) Atrial and ventricular average Ca^2+^ levels (Twitch-4 emission ratio) and fractional area change (FAC) in control larvae (*n* = 18), in larvae during incubation with 75 µM *para*-aminoblebbistatin (PAB 1 h, *n* = 8, and PAB 2 h, *n* = 22), and in larvae during washout after 2 h of PAB treatment (washout 2 h, *n* = 20, and washout 4 h, *n* = 20). Statistical analysis was performed using a one-way ANOVA test with Dunnett’s multiple comparisons post-test. Data are shown as the mean ± SD (** *p* < 0.01, *** *p* < 0.001, **** *p* < 0.0001). (**B**) Inverse correlation between heart contraction and Ca^2+^ levels (Twitch-4 emission ratio) of individual larvae before (control), after incubation with 75 µM PAB (1 or 2 h), and after washout of PAB (2 or 4 h). A linear regression test was performed, and the coefficient of correlation (R^2^) was calculated.

The effect of blebbistatin on heart contraction has been found to be concentration-dependent and reversible upon washout of the drug [18]. We assessed the reversibility of the PAB effects in 3 dpf *Tg(myl7:Twitch-4)* zebrafish larvae. We measured Ca^2+^ levels and contraction strength in larvae treated with 75 µM PAB for 1 h or 2 h, and 2 or 4 h after drug washout. Incubation with PAB for 1 h decreased FAC and raised Ca^2+^ levels, whereas at 2 h, heart contraction was almost completely abrogated and Ca^2+^ levels had increased further. Upon washout of PAB, heart contraction partially recovered: the fractional area change (FAC) reverted to 0.89 and 0.73 of its basal value in the atrium and ventricle, respectively, after 4 h of PAB washout (Figure 5A). Likewise, the Ca^2+^ levels (the average of the systolic and diastolic Twitch-4 emission ratio) decreased in the atrium and ventricle after PAB washout. Furthermore, heart contraction and Ca^2+^ levels correlated with each other during incubation and washout of PAB (Figure 5B). These results support the reversibility of PAB effects upon washout and show that the increase in Ca^2+^ levels is concomitant with the reduction in the force of contraction.

## 4. Discussion

In this study, we used various gene-encoded Ca^2+^ biosensors in our experiments: the fluorescent Twitch-4 [20] and mCyRFP1-GCaMP6f [10], and bioluminescent GFP-aequorin [21]. The aim was to ensure that the estimated Ca^2+^ levels in the larval heart were independent of the biosensor employed. In FRET biosensors like Twitch-4, Ca^2+^ decreases the donor emission and enhances the acceptor emission, so the ratio of FRET to donor fluorescence enhances the response. Twitch-4 has a mutagenized troponin C from the toadfish *Opsanus tau* as the Ca^2+^-binding domain, and its apparent K*d* for Ca^2+^ is 2.8 µM [20]. We recently reported mCyRFP1-GCaMP6f, a ratiometric Ca^2+^ biosensor not based on FRET [10]. GCaMP6f is an intensiometric indicator [22] with calmodulin as the Ca^2+^-binding moiety and a K*d* of 375 nM. Since GCaMP6f has a single emission wavelength and no spectral shift upon binding Ca^2+^, the red fluorescent protein mCyRFP1 was molecularly fused as a Ca^2+^-insensitive reference fluorophore [10]. mCyRFP1 is a long-Stokes-shift fluorescent protein excited at the same wavelength as GCaMP6f but emitting in the red range [23]. In PAB-treated larvae and *tnnt2a* morphant larvae, similar changes in CaT frequency and Ca^2+^ levels were seen regardless of the biosensor employed.

Here, we showed that bradycardia resulted from halting heart contraction by either genetic means or by the myosin II inhibitor PAB (Figure 2, Figure 3 and Figure 4). Umemoto et al. coinjected zebrafish embryos with a control MO labeled with the fluorophore lissamine together with a *tnnt2* MO [24]. The fluorescence of embryos, which correlated inversely with the expression levels of tnnt2a in the morphants, allowed for the selection of embryos with a moderate reduction in cardiac function. Zebrafish partially depleted of cardiac troponin T were found to have a decreased heart rate (as in our study) and impaired cardiac contraction with both systolic and diastolic dysfunction.

The spontaneous diastolic depolarization rate in the sinoatrial node determines the heart rate. The speed of membrane depolarization is a product of at least three oscillatory mechanisms: the membrane (voltage) clock, the Ca^2+^ clock, and the mechanics clock [25]. In vivo, all three mechanisms likely contribute to setting the heart rate, a robust but flexible system that helps match the cardiac output to the organism’s varying needs. HCN channels are related to the membrane clock, and their activity can be modulated by the autonomous system, circulating hormones, and, possibly, by cell stretching. Spontaneous and rhythmic release of Ca^2+^ from the sarcoplasmic reticulum stores activates the NCX, depolarizing the membrane, the so-called Ca^2+^ clock. The mechanics clock is thought to couple the heart rate to changes in the hemodynamic load on a beat-to-beat basis. The molecular mechanism involves cation-nonselective stretch-activated channels (SAC_NS_). In beating hearts, rhythmic atrial stretch is involved in setting the basal heart rate. Thus, mammals and other vertebrates display a compensatory rise in heart rate when venous return is increased, or simply by distension of the right atrium, the well-known Bainbridge effect [26]. This effect has also been demonstrated in zebrafish and may be mediated by reflexes (extrinsic regulation) or by intrinsic mechanisms (cardiac nervous system, local mediators, or mechanical stretch) [27]. It was found in that study that stretch causes a rapid increase in the beating rate in isolated preparations of the sinoatrial ring. We propose that arresting contraction in our experiments by either *tnnt2a* MO or PAB causes bradycardia due to the lack of such mechanical stimulation.

In our results, systolic and diastolic Ca^2+^ levels and CaT increased in both *tnnt2a* morphants and PAB-treated larvae, suggesting that halting heart motion alters cardiac Ca^2+^ homeostasis. Several mechanisms could be at play. The arterial baroreflex is the main short-term regulator of arterial blood pressure in vertebrates. In PAB-treated or *tnnt2a* morphant zebrafish, the drop in cardiac output and arterial pressure could be triggering this reflex, with a compensatory increase in adrenergic input (comprising Ca^2+^ influx through L-type Ca^2+^ channels). Although the adrenergic system is active in 3 dpf zebrafish [10], to our knowledge, the baroreceptor reflex has not been characterized in this organism but exists in other teleosts [28].

The observed increase in cardiac Ca^2+^ levels and CaT amplitude in the two models could also be due to an extended plateau phase of the action potential, enhancing L-type Ca^2+^ current [29]. Working cardiomyocytes possess SAC_NS_, with a reversal potential about halfway between resting voltage and peak depolarization [25]. During the action potential plateau, the stretch-activated current through SAC_NS_ accelerates repolarization; thus, the lack of stretch in arrested larval hearts (Figure 1C) would result in a longer plateau with increased Ca^2+^ influx.

We think the key perturbation altering CaT was the elevation of the diastolic Ca^2+^ level: as it increases, the buffering power of endogenous Ca^2+^ buffers decreases as they are more saturated. Thus, the same or even a smaller increase in total Ca^2+^ would result in a larger systolic Ca^2+^ level and CaT amplitude [30]. It seems that a feedback mechanism is in place: the lack of contraction (by *tnnt2a* MO or PAB) results in higher Ca^2+^ levels in an attempt to increase the force of contraction. The rise in diastolic Ca^2+^ could result from increased L-type Ca^2+^ current or inhibition of the NCX (by increased cytosolic Na^+^ content). A decrease in the SERCA activity would also raise diastolic Ca^2+^. In addition, there are poorly characterized background Ca^2+^ entry mechanisms in cardiomyocytes, independent of electrical stimulation [30]. They possibly involve connexin hemichannels or transient receptor potential channels (like TRPV2). Arresting heart motion could increase background Ca^2+^ influx, raising diastolic Ca^2+^ levels. Unfortunately, dissecting these potential mechanisms is difficult in the larval heart in vivo.

Zebrafish *tnnt2a* morphants and mutants (silent heart, *sih*) display complete cardiac arrest and sarcomere disarray [2]. In addition to a lack of tnnt2a, these morphants showed reduced levels of ∝-tropomyosin and cardiac troponins C and I, components of the Ca^2+^-sensitive regulatory complex, possibly reducing the endogenous buffering capacity. These morphants also presented defects in trabeculation and valve formation [31,32]. Since trabeculae act as an early conduction system in the larval ventricle, the atrioventricular conduction blocks observed in some *tnnt2a* morphant hearts at 3 and 5 dpf (Figure 1F,G) could be due to a malfunction of ventricular electrical excitation or a block in the atrioventricular canal due to altered action potentials [33].

In conclusion, in our results, *tnnt2a* MO and PAB caused similar alterations in heart rate and Ca^2+^ levels, as measured with orthogonal techniques (two fluorescent biosensors and a bioluminescent biosensor). Thus, suppression of heart contraction was the likely cause of the bradycardia and altered Ca^2+^ homeostasis through changes in adrenergic input, action potential duration, Ca^2+^ currents, stretch-activated channels, and/or endogenous Ca^2+^ buffering. Furthermore, the possibility exists that different mechanisms are engaged in the *tnnt2a* MO and PAB experiments. Nevertheless, the fact remains that both treatments interfered with cardiomyocyte Ca^2+^ levels in vivo. Thus, cardiac contraction appears to be an important regulator of systolic and diastolic Ca^2+^ levels. In contrast to electromechanically uncoupled hearts, optical mapping studies in unconstrained contracting hearts allow the study of interactions between contraction, electrophysiology, and metabolism, and enhance the translational applicability of results [10,19,33,34].

## Data Availability

The raw data supporting the conclusions of this article will be made available by the authors on request.

## Supplementary Materials

The following supporting information can be downloaded at: https://www.mdpi.com/article/10.3390/bios14050219/s1, Video S1: Sibling zebrafish larva at 3 dpf; Video S2: tnnt2a morphant zebrafish larva at 3 dpf; Video S3: PAB-treated zebrafish larva at 3 dpf.
