# Supplementary figures and images for: Suppression of Contraction Raises Calcium Ion Levels in the Heart of Zebrafish Larvae
Source: Biosensors (Basel). 2024 Apr 27;14(5):219. doi: 10.3390/bios14050219 (PMC11118826; doi:10.3390/bios14050219)

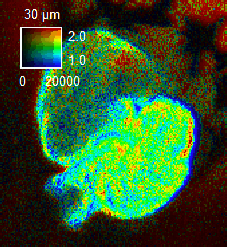

Supplement: Supplementary file 1 [file biosensors-14-00219-s001.zip › Video S1_Siblings.gif]

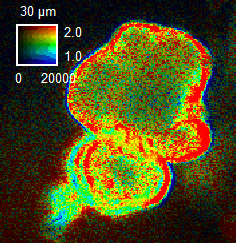

Supplement: Supplementary file 1 [file biosensors-14-00219-s001.zip › Video S2_Tnnt2a MO.gif]

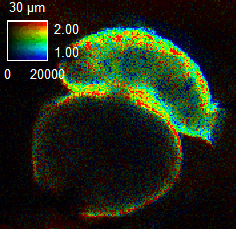

Supplement: Supplementary file 1 [file biosensors-14-00219-s001.zip › Video S3_PAB.gif]
